# Impact of Hospital Design on Acutely Unwell Patients with Dementia

**DOI:** 10.3390/geriatrics2010004

**Published:** 2017-01-12

**Authors:** Caitlin Young, Chris Edwards, Inderpal Singh

**Affiliations:** 1 Medical student, School of Medicine, Cardiff University, Cardiff CF10 3AT, UK; YoungC9@cardiff.ac.uk; 2 Consultant Clinical Scientist, Department of Dermatology, Aneurin Bevan University Health Board, Newport NP20 4SZ, UK; Chris.Edwards3@wales.nhs.uk; 3 Consultant Geriatrician, Department of Geriatric Medicine, Ysbyty Ystrad Fawr, Aneurin Bevan University Health Board, Wales CF82 7EP, UK

**Keywords:** dementia, single rooms, multi-bedded wards, older people, falls, clinical outcomes

## Abstract

Increasing emphasis on patient privacy and satisfaction has seen more 100% single-room hospitals opened across the UK. Few studies have addressed the impact of these new hospital designs (single rooms) on clinical outcomes specifically for acutely unwell frail patients with dementia. The objective of this study was to profile and compare the clinical outcomes of acutely unwell patients with dementia admitted to two different hospital environments. This prospective observation study was conducted for 100 dementia patients admitted at Ysbyty Ystrad Fawr (hospital with 100% single rooms) and Royal Gwent Hospital (traditional multi-bed wards) under the same University Health Board. The length of stay (LoS) was significantly longer for patients admitted to single rooms. The clinical profile of the patients was similar in both hospitals and has no association with LoS. There was no significant difference in terms of incidence of inpatient falls, fall-related injury, discharge to a new care home, 30-day readmission, or mortality. The single room environment appears to influence LoS, as previously reported; however, following the introduction of quality improvement initiatives to prevent inpatient falls, single rooms do not appear to be associated with higher inpatient fall incidence. We propose more research to understand the relationship between single rooms and LoS.

## 1. Introduction

Hospitalisation is hazardous for frail older people and particularly for those with dementia [1,2,3]. Dementia-friendly environments have been proposed to promote patient well-being, mobility, independence, and meaningful interaction between other patients and staff/family; however, there has been little emphasis on single room design [4]. Single room environments do provide enhanced, dignified care [5,6,7,8], but at the expense of higher perceived loneliness [9]. Hospitalisation is associated with higher adverse outcomes for those with dementia [3,10,11] and people with dementia are at a 2.5 times higher risk of inpatient falls [12]. A recent prospective cohort study reported a higher incidence of inpatient falls in dementia patients occupying single rooms as compared to those in traditional multi-bedded wards (MBWs) [13]. Furthermore, people with dementia are at increased risk of sustaining a serious injury following a fall compared to those without dementia, the incidence of fracture being three times higher amongst fallers with dementia [14,15]. People with dementia are at higher risk of delirium and a recent meta-analysis has shown an association between delirium and poor outcomes for older patients in the hospital [16]. 

Hospital design may influence clinical outcomes of acutely ill frail people. Few studies have examined dementia outcomes in the general hospital setting hospital and none have compared the clinical outcomes of acutely unwell patients with dementia in two different hospital environments. The aims of this study were to broadly describe acutely unwell patients with dementia admitted to two different hospitals environments—single rooms and traditional MBW—and to study the clinical outcomes and predictors of adverse outcomes in these two environments. 

## 2. Materials and Methods

### 2.1. Study Design

A prospective observational cohort study was conducted to complete descriptive analysis of the patients in each hospital site and investigate the impact of hospital environment on clinical outcomes.

### 2.2. Setting

Royal Gwent Hospital (RGH) is a 774-bed district general NHS hospital in Newport, South Wales. Ysbyty Ystrad Fawr (YYF) is a new 269 bed local general NHS hospital Ystrad Mynach, South Wales commissioned in 2011. YYF provides 100% single en suite rooms. YYF was designed in line with the current trend towards single rooms, which are thought to offer patients more control over their immediate environment, privacy, and minimise the spread of infection (6). Both hospitals admit acute and subacute patients and there are no socioeconomic differences between the locations of the two hospitals. Both hospitals operate under the Aneurin Bevan University Health Board (ABUHB). 

### 2.3. Data and Measurements

One hundred patients were prospectively observed, 50 at YYF (single rooms) and 50 at RGH (multi-bedded wards—MBWs). Inclusion criteria were any older patient with diagnosed dementia admitted with an acute illness. Patients with a terminal illness and pre-planned admissions were excluded. Patients were recruited between May and July 2016 and outcome analysis was updated until the end of November 2016. Data were transcribed from medical, nursing, occupational therapy, and physiotherapy paper notes and Welsh Clinical Portal, anonymised and incorporated into one data-collection form per participant by the data collector. 

Data collected on admission for each individual included age, sex, source of admission, reason for admission, Montreal Cognitive Assessment score (MoCA) [17], carer support (formal or informal), Charlson Comorbidity score [18], Barthel Index (BI) Activities of Daily Living (ADLs) [19] two weeks pre-admission and on admission, extended ADLs [20], anti-psychotic/sedative prescriptions, hearing impairment, visual impairment, MUST malnutrition score [21], pain on admission, depression, delirium, Confusion Assessment Method (CAM) score [22], and Behavioural and Psychological Symptoms of Dementia (BPSD) [23]. The cause of admission was recorded for each patient. 

To investigate the effect of the hospital environment, we collected clinical outcome data for individual patients including the length of stay (LoS), discharge destination, inpatient mortality, 30- day readmission, and inpatient falls and associated fracture. The index admission was defined as any one episode of admission until discharge from the health board or until death. Inpatient falls were defined using the same criteria as a previous study of inpatient falls in RGH and YYF [13]; an incident in which a patient came to rest on the floor or lower surface, with or without a loss of consciousness. Fall data were obtained from DATIX, a web-based patient safety software for reporting of incidents. 

### 2.4. Statistical Analysis 

Statistical analysis was performed using IBM SPSS 23 [24]. Means ± standard deviations were calculated for baseline characteristics of patients at both hospitals. Calculations of proportions were made for patients admitted from different residences, prescribed antipsychotics/sedatives and with visual and hearing impairment, pain, depression, delirium, and displaying BPSD. *t*-tests were used to compare mean baseline characteristics of patients in both sites, and chi-square tests were used to compare categorical variables . An ANOVA test was performed to assess the effect of independent variables (i.e., baseline characteristics) on output dependent variables (clinical outcomes). Correlations between LoS and continuous baseline variables were assessed with the Pearson R statistic and associated *p*-values. *p*-values ≤0.05 were taken to be statistically significant. 

### 2.5. Ethical Approval 

This study is classed as a service evaluation project according to the Health Research Authority decision tool. Our study sought to measure the current standard of care in ABUHB, comparing single rooms and MBWs. All documents to be used in data collection were submitted to the ABUHB Research and Development (R&D) department, which approved the project as an observational service evaluation with no requirement for ethical approval. All demographic and outcome data incorporated into the study are routinely documented by ABUHB and recorded in patient notes. Furthermore, no identifiable data besides sex, date of birth, and hospital identification number were recorded. No identifiable patient information has been or will be shared. Patient consent was still taken where possible in writing or verbally.

## 3. Results

A total of 50 patients were observed at each hospital. There was no statistically significant difference in the mean age of patients admitted to single rooms (83.4 ± 8.4, range = 53–95 years) or MBWs (82.8 ± 8.4, range = 59–96 years, *p* = 0.73). There were a higher proportion of women in each hospital site (single rooms = 30/50; MBWs = 31/50, *p* = 0.84).

Most patients (73%, *n* = 73/100) were admitted from their own homes. Other sources of admission included residential homes (14%), nursing homes (3%), EMI residential (2%), EMI nursing homes (2%), warden-run sheltered accommodation (4%), and respite care (1%). All patients received some form of social and/or medical community care, be this formal, informal, or both. The majority of patients were in receipt of a formal package of care in both single rooms (68%, *n* = 34/50) and MBWs (72%, *n* = 37/50 respectively). Significantly more patients in single rooms (88%, *n* = 44/50) were admitted from their own homes compared to those in MBWs (58%, *n* = 29/50) (*p* = 0.007) and also had significantly better pre-admission levels of independence as measured by pre-admission BI. Besides the source of admission, there were no significant differences in baseline characteristics of acutely unwell patients with dementia admitted either to single rooms or MBWs (Table 1).

The reasons for acute admission varied widely, though falls were the most common reason for admission to both sites, with a significantly higher proportion of patients with falls as a presenting complaint admitted to single rooms (56%, *n* = 28/50) as compared to MBWs (24%, *n* = 12/50, *p* = 0.01). In addition, a significantly higher proportion of patients admitted to single rooms had fall-related fractures in the community (32%, *n* = 9/28) as compared to MBWs (8%, *n* = 1/12, *p* = 0.008). Sepsis, urinary tract infections, loss of consciousness, and confusion were other common presenting complaints (Table 2).

Patients who had been transferred across two sites and therefore exposed to both environments (single rooms =7 and MBWs = 4) were excluded from outcome analysis. The majority (84%, *n* = 75/89) of acutely unwell dementia patients (single rooms = 39, MBWs = 36) were discharged from hospital. Of these, a similar number were successfully discharged to their original residence (*p* = 0.132) (Table 3). 14 single room patients and 7 MBW patients were discharged to a new care home and there was no significant difference (*p* = 0.092). 1 MBW patient remained in hospital at the time of writing. 

The mean total LoS was significantly higher for patients discharged from single rooms (62.23 ± 41.79) as compared to those from MBWs (42.47 ± 40.50 days, *p* = 0.027). 

Six patients experienced inpatient fall (IF) in each hospital site (*p* = 0.572), with a total of incidence of 12 inpatient falls in single rooms and 8 in MBWs (*p* = 0.175). There were no significant differences in the number of recurrent fallers (*p* = 0.629). There were no significant differences in inpatient mortality (*p* = 0.21) or 30-day readmission rate (*p* = 0.335). 

In this study, we observed too few adverse outcomes of inpatient falls, inpatient deaths, or discharge to the new care home to reliably test for association with any of the baseline patient variables recorded on admission. Therefore, only sub-analysis to examine factors that could predict the higher length of stay was completed. Advancing age, the presence of BPSD, and admission to a single room were the only factors found to be associated with increased LoS (*p* = 0.007, 0.017, 0.027, respectively). 

## 4. Discussion

Most attention to hospital environments has centred on patient satisfaction, quality of sleep, privacy, and dignity [7,25,26]. Few studies have sought to empirically address the impact of hospital design on patient safety and clinical outcomes. 

Studies have reported a higher incidence of IF and other associated adverse outcomes in single rooms [27,28,29,30]. An evaluation of the impact of single rooms on staff and patient experience, safety, and cost, found that a majority of patients expressed a preference for private rooms and no differences were observed in patient safety, though nursing staff expressed concerns over loss of the wider patient surveillance [26]. Other literature has focused on the effect of single rooms in acute settings [8], predominantly addressing the impact on younger patients and those without cognitive impairment. This study evaluates the impact of hospital design on acutely unwell frail, older patients with dementia, many of whom had prolonged hospital stays due to acute illness and associated impact of hospitalisation. There is very little literature concentrating specifically on outcomes for patients such as these in different ward environments, namely, single rooms and MBWs.

In this study, it was observed that acutely unwell older people with dementia admitted to single rooms and MBWs had a largely similar demographic profile and clinical characteristics. However, acute patients with dementia admitted to single rooms had a significantly longer LoS than those admitted to traditional MBWs. Besides LoS, no other significant differences were observed in clinical outcomes between patients in single rooms and those in MBWs. It was observed that patients admitted to single rooms have clear documentation of the MoCA and MUST scores, and only one patient admitted to MBWs has a recorded MoCA score. However, this could be observer bias.

Our observation of increased LoS in single rooms builds upon similar findings reported from a previous study which also reported a higher incidence of inpatient falls in patients with dementia in single rooms as compared to MBWs [13]. Interestingly, we found no significant difference in the incidence of IF between single rooms and MBWs. This could be due to the introduction of quality initiatives to minimise inpatient falls in single rooms [30]. A systematic nurse training programme on the understanding and correct use of existing multifactorial falls risk assessment (FRA) tools in the single room hospital has demonstrated a significant and sustained reduction in the mean incidence of IF [30]. Similar rates of IF between single rooms and MBWs may therefore be looked upon in an encouraging light.

In the previous study, discrepancies in LoS between single rooms and MBWs were attributed to a possible cause and effect relationship with rates of IF [13]. We found no significant differences in the incidence of IF, suggesting that there are other possible factors resulting in comparatively longer LoS in single rooms. 

We studied potential confounding factors such as age, delirium, pain on admission, depression, and severity of dementia or associated BPSD. We found no significant association between delirium, pain on admission, or clinical severity of dementia and LoS in the two hospital settings. We did, however, observe associations between LoS and advancing age. The information on BPSD was mentioned in only half of the patients (49/100). There was no significant difference in the frequency of BPSD in patients admitted to either hospital. The presence of BPSD was associated with a significantly longer length of stay in either hospital. We acknowledge due to limited available data on details of BPSD, a detailed evaluation was not possible and due to the low power of the study, these are weak associations. 

A significantly higher number of patients were admitted to single rooms having sustained a fall and fall-related fractures in the community, which may contribute towards a longer LoS in single rooms. However, sub-analysis showed no statistically significant association between fracture on admission to the single room and LoS (*p* = 0.60). A significantly higher number of patients were admitted from their own homes in single rooms, and there could be a barrier in accessing community care or a higher threshold to gain a pre-admission level of function. In comparison, a greater proportion of patients were admitted to MBWs from care homes, which could in part explain the comparatively shorter LoS in MBWs since there may be a lower threshold of functional independence prior to discharge. In addition, though not significantly different, a higher proportion of patients were discharged to a new care home from single rooms.Since it is more time-consuming to find a new care home for an older person with dementia to meet all care needs, this might be another reason for the higher length of stay in single rooms, alongside other unexplored factors. 

Our study has several strengths. Firstly, we are not aware of any study that has reported the impact of hospital design on acutely unwell patients with dementia prospectively. Furthermore, we have described acutely unwell patients with dementia comprehensively and studied the clinical outcomes, measuring potentially confounding factors. This study could lead to a more detailed multi-centre study. 

Though our results are interesting, we acknowledge that our study is limited by its small size. Neither participation rate nor the total number of dementia patients admitted to the Health Board were measured. In addition, we have not studied regional differences in the provision of intermediate or community resource teams, which facilitate discharge from hospitals. Dementia care strategies have been in place in single rooms, but we acknowledge that we did not study the details of similar strategies in MBWs. Methods of data collection may also be considered a limitation, as data were extracted from patient notes; thus, poor documentation of clinical information resulted in limited availability of data. This limits the power of the study, particularly for confounding factors. For example, MoCA scores were not well recorded for patients admitted to MBWs and the severity of cognitive impairment was based on clinical notes; therefore, differences in cognitive status could explain the prolonged LoS in single rooms. In addition, we have not measured frailty status using phenotypic frailty indicators [31] or frailty index of accumulated deficits, which have shown associations with adverse outcomes [32]. Though not statistically significant, gender differences may also bias the results. Finally, our observational study took place across two hospitals within the same health board in South Wales (UK); the results may not be generalisable to the population as a whole. 

Our study focused on the impact of hospital environments on quantitative, measurable clinical outcomes. As such, we did not explore the experiences of older people with dementia in single rooms. In light of the reduced social interaction and relative isolation reported by some older adults in single rooms, it is possible that some older people with dementia may not express a preference for single-room hospital accommodation [9,33]. Numerous personal, cultural, socioeconomic, and medical factors may affect preferences, and those of older people with dementia should be explored further [34]. Prompt assessments by the older adult psychiatry liaison service for acutely unwell older people with mental health problems are not only cost-effective by reducing length of stay but also improve clinical outcomes. This trend has been observed particularly in older adults [35,36,37]. We propose a larger prospective study to confirm our findings and measure the impact of hospital design on clinical outcomes of acutely unwell patients with dementia. Further research to explain the longer length of stay in single rooms is also warranted. 

## 5. Conclusions

This observational study suggests hospital environments may affect clinical outcomes, with a significantly higher length of stay for acutely unwell patients with dementia admitted to single rooms as compared to those in MBWs. However, no other significant differences were observed in clinical outcomes in terms of inpatient mortality, inpatient falls, discharge to a new care home, or 30-day readmission. 

## Figures and Tables

**Table 1:** Clinical characteristics of the older people with dementia admitted to hospital.

	YYF (Single Rooms)	RGH (Multi-Bedded Wards)	*p*
No. of patients	50	50	
Age, mean ± SD	83.4 ± 8.4	82.8 ± 8.4	0.73
Female, n/N (%)	30/50 (60)	31/50 (62)	0.84
Place of original residence			
Private residence, n/N (%)	44/50 (88)	29/50 (58)	0.007
Residential home, n/N (%)	3/50 (6)	11/50 (22)	0.021
Nursing home, n/N (%)	0/50 (0)	3/50 (6)	0.8
EMI residential home, n/N (%)	0/50 (0)	2/50 (4)	0.15
EMI nursing home, n/N (%)	0/50 (0)	2/50 (4)	0.15
Other, n/N (%)	3/50(6)	3/50 (6)	1.0
MoCA score, mean ± SD	11.6 ± 4.9	-	0.62
Carer support			
Formal, n/N (%)	34/50 (68)	37/50 (74)	0.5
Informal, n/N (%)	16/50 (32)	11/50 (22)	0.26
Charlson comorbidity burden, mean ± SD	5.0 ± 1.4	5.1 ± 1.1	0.69
ADLs 2 weeks pre-admission, mean ± SD	12.0 ± 4.6	7.7 ± 5.8	0.05
ADLs on admission, mean ± SD	7.7 ± 5.2	4.5 ± 3.7	0.25
Number of extended ADLs requiring assistance, mean ± SD	3.5 ± 1.1	3.2 ± 1.1	0.42
Number of medications on admission, mean ± SD	8.2 ± 4.1	9.7 ± 4.1	0.09
Patients prescribed antipsychotic or sedative, n/N (%)	3/50 (6)	8/50 (16)	0.10
Patients with hearing impairment, n/N (%)	15/50 (30)	6/50 (12)	0.38
Patients with visual impairment, n/N (%)	20/50 (40)	5/50(10)	0.072
MUST malnutrition score, mean ± SD	0.75 ± 0.96	-	
Patients with pain on admission, n/N (%)	19/50 (38)	15/50 (30)	0.42
Patients with depression, n/N (%)	13/50 (26)	17/50 (34)	0.42
Patients with delirium, n/N (%)	21/50 (42)	18/50 (36)	0.43
Patients with BPSD, n/N (%)	13/50 (26)	14/50 (28)	0.24

**Table 2:** Cause of admission for patients in single rooms and multi-bedded wards (MBWs).

	YYF (Single Rooms)	RGH (Multi-Bedded Wards)
Fall, n/N (%)	28/50 (52)	12/50 (24)
Confusion, n/N (%)	6/50 (12)	3/50 (6)
Sepsis, n/N (%)	3/50 (6)	8/50 (16)
UTI, n/N (%)	6/50 (12)	5/50 (10)
Stroke, n/N (%)	4/50 (8)	0/50 (0)
Loss of consciousness, n/N (%)	0/50 (0)	3/50 (6)
Pneumonia, n/N (%)	1/50 (2)	3/50 (6)
SOB, n/N (%)	0/50 (0)	3/50 (6)
MSK pain, n/N (%)	1/50 (2)	1/50 (2)
Chest pain, n/N (%)	0/50 (0)	3/50 (6)
Constipation, n/N (%)	1/50 (2)	0/50 (0)
D&V, n/N (%)	0/50 (0)	1/50 (2)
Reduced consciousness, n/N (%)	0/50 (0)	1/50 (2)
Functional decline, n/N (%)	0/50 (0)	1/50 (2)
Pyrexia, n/N (%)	0/50 (0)	1/50 (2)
Seizure, n/N (%)	0/50 (0)	1/50 (2)
Urinary retention, n/N (%)	0/50 (0)	1/50 (2)
Alcohol withdrawal, n/N (%)	0/50 (0)	1/50 (2)
CHF, n/N (%)	0/50 (0)	2/50 (4)

**Table 3:** Summary of clinical outcome of acutely unwell patients with dementia.

	YYF (Single Rooms)	RGH (Multi-Bedded Wards)	*p*
No. of patients excluded, n/N (%)	7/50 (14)	4/50 (8)	0.538
No. of patients discharged, n/N (%)	39/43 (91)	36/46 (78)	0.133
Discharge to original residence, n/N (%)	25/39 (64)	29/36 (80)	0.132
Discharge to a new care home, n/N (%)	14/39 (36)	7/36 (19)	0.092
No. of patients remaining in hospital, n/N (%)	0/43 (0)	1/46 (2)	0.517
In-patient mortality, n/N (%)	4/43 (9)	9/46 (20)	0.133
Length of Stay, mean ± SD (days)	62.23 ± 41.79	42.47 ± 40.50	0.027
No. of in-patients who fell, n/N (%)	6/43 (14)	6/46 (13)	0.572
Total no. of falls, n/N (%)	12	8	0.175
No. of of fractures, n/N (%)	0 (0.0)	0 (0.0)	-
Readmitted within 30 days, n/N (%)	9/43 (21)	7/46 (15)	0.335
